# Low-Cost, Scalable Classroom-Based Approach to Promoting Physical Activity in Preschool Children

**DOI:** 10.21767/2572-5394.10008

**Published:** 2016-05-09

**Authors:** Shelly K McCrady-Spitzer, Vanessa Sagdalen, Chinmay U Manohar, James A Levine

**Affiliations:** 1 Department of Endocrinology, Mayo Clinic, Rochester, MN, USA; 2 Department of Early Childhood Family Education & School Readiness, Plainview-Elgin-Millville Community Schools ISD 2899, Plainview, MN, USA; 3 Department of Research & Technology Innovation, Roche Diabetes Care Inc, Indianapolis, IN, USA; 4 Obesity Solutions, Mayo Clinic Arizona and Arizona State University, Scottsdale, AZ, USA

## Abstract

**Background:**

This study examined the impact of short activity breaks in preschool children. The hypotheses were that preschool children receiving three five-minute activity breaks per day would increase (a) school time physical activity and (b) education scores compared to a control group not receiving the intervention.

**Methods:**

For 8 weeks, the Intervention Group (n = 13) incorporated three 5-minute activity breaks into their classroom time while the Control Group (n = 12) did not incorporate the activity breaks. Physical activity was measured using a triaxial accelerometer. Education was assessed using standardized methods.

**Findings:**

After 8 weeks, the preschool children in the Intervention Group increased their school time physical activity from 11,641 ± (SD) 1,368 Acceleration Units (AU)/ hour to 16,058 ± 2,253 AU/hour (P < 0.001). The children in the control group did not increase their physical activity (11,379 ± 2,427 cf 11,624 ± 2,441; ns). Students in the Intervention Group improved their education scores more than students in the control group (18 ± 12 cf 8 ± 7 points, P = 0.01); Letter Recognition improved in particular (9 ± 6 cf 2 ± 4 points, P = 0.001).

**Conclusions:**

The incorporation of three 5-minute activity breaks was associated with increased school time physical activity and improved learning.

## Introduction

Low levels of daily physical activity in children are commonplace and contribute to metabolic disorders [1] and childhood obesity [2–4]. School-based daily activity is threatened across the age span by decreases in physical activity lessons and diminished resources for sports and recreational equipment [5,6].

A child’s daily activity can be promoted at home, in the community, and at school [5,7]. The earlier one can intervene to promote physical activity in children, the greater the potential long-term health benefits [8–11]. Although it is challenging to design, deploy and test interventions in very small children, preschool children are at a particularly important age to promote physical activity [11]. Data suggest that the brain is most plastic at a young age and becomes less adaptable as growth and development occur [12–15].

We conducted a study in preschool child to evaluate the impact of three short (five minutes) activity breaks per school day. We vigorously measured the impact of the program using validated research grade accelerometers. The primary hypothesis was that preschool children exposed to three 5- minute activity breaks per day would increase the overall school time physical activity compared to a control group not receiving the intervention. The secondary hypothesis was that children receiving the three 5-minute activity breaks per school day would show improved literacy and numeracy scores compared to the control group. This approach is attractive because it is inexpensive, does not require any facilities or equipment and can readily be disseminated.

## Methods

### Setting

The study took place in Plainview-Elgin-Millville School District, which is a public, rural, community school in the 55964 zip code with enrollment at 1,450 students. Of the children, 29% qualify for the Free/Reduced Lunch Program, 12% require special education, 10% are from recognized minorities, and 6% are open-enrolled. There are 153 staff, 106 of those are teachers. The School Readiness Preschool Program, where the study was conducted, is located in the PK-3 building. The school is in Wabasha County, MN Federal Information Processing Standards Code 27157.

### Students

Participants in this study were enrolled in School Readiness Preschool which consists of two three-hour classes per week (total educational time is 6 hours/week). The preschool children ranged from 3–5 years old.

There were two groups of children: one group attended on Mondays and Wednesdays. The other group attended on Tuesdays and Thursdays. The same teacher taught both groups of children. By closed envelope assignation; the Monday/ Wednesday group was designated as the Control Group and the Tuesday/Thursday group was designated the Intervention Group.

### Protocol

The study was approved by the Hamline University School of Education. Children provided verbal agreement, and parents, caregivers, provided informed written consent.

The protocol was 10 weeks long. The first two weeks were for collection of baseline measurements of school time physical activity, body weight & height and educational attainment.

For the last eight weeks, the Intervention Group received the activity-enhancing program plus the standard curriculum, whereas the Control Group received the standard curriculum alone.

During this study, both classes followed the same curriculum, academic activities and gym time. The same materials were taught to both groups in the same weeks.

### The intervention

The intervention was to incorporate three 5-minute activity-promoting tasks per school day; representing an extra 15 minutes of activity per day. The control group did not receive the activity tasks.

The intervention was carried out using an empty coffee can into which multiple small pieces of paper were placed – each containing an activity task. The activity tasks included: running in place, frog jumps, jumping up and down, sit-ups, wiggling, and movements used to pretend the students were ice skating, playing basketball, moving like animals, and boxing. During the 5-minute activity task, multiple slips of activities were drawn to keep the students engaged.

Each activity task was conducted using the ‘follow-the-leader style’ or the ‘copycat method’. ‘Follow-the-leader style’ is where the students stood in line and followed the teacher around the room performing the task. The ‘copycat method’ is where the teacher models the activity and then the students copy her.

To compensate for the 15 minutes of extra activity during the class, the Intervention Group had a shorter free choice time at the start of the day and a shorter large motor free choice time at the end of class.

## Measurements

### Height and Weight

Height and weight were measured at baseline and at the end of the 8-week intervention by trained personnel using calibrated scales (Detecto 438 Webb City, MO) and a stadiometer (Detecto 438 Webb City, MO) using established protocols [16,17]. Children wore light clothing, and shoes were removed.

### School time physical activity

Physical activity was measured at baseline and after 4 and 8 weeks of the Intervention/Control periods. Physical activity was measured using validated triaxial accelerometers (Modular Signal Recorder [MSR] 145 mini data logger series Omni Instruments, Dundee, Scotland 18 x 14 x 62 mm [18 g]).

The accelerometer was fixed to the center of the child’s back using an elastic belt. For each 5-day accelerometer measurement period, research staff attached an individually fitted elastic accelerometer belt to every student at the beginning of the school day, and removed it at the end of the school day.

MSR data were gathered at 20 Hz (20 acceleration x, y, z axis measurements per second), with an accelerometer measurement range of 2 g, which is optimum for daily activity in children [18,19]. The MSR stored > 2 million data points, was housed in a waterproof silicon sheath, was powered by a lithium-polymer battery, and was charged daily.

The MSR compares favorably to state-of-the-art activity/ posture measurement systems, (a) reliably distinguishes sedentary and walking activity with ½-mph walking speed increments; (b) shows excellent intraclass correlation coefficient (R^2^ > 0.95) compared to gold standard measurements; (c) demonstrates excellent repeatability sequential accelerometer increases with increased walking velocity (R^2^ > 0.95); where velocity [in mph] = 0.0134 AU/min – 2.61 and (d) energy expenditure (R^2^ > 0.94) [20].

### Educational attainment

Students were tested individually on their number and letter recognition [21] at baseline and after 8-weeks of the Intervention or Control period. Letter recognition was tested for both the capital and lower case letters of the alphabet including the sound identification of each individual letter, and their 0–10 number identification.

They were given one chance for each letter and number. If they were correct, they received 1 point. If a student answered incorrectly but corrected themselves within 5 seconds without prompting from the examiner they would receive 1 point. If the student responded incorrectly or with no answer after 10 seconds they received no points for the question. The Overall Education Score was the sum of the Letters Score (maximum 52), plus the Sounds Score (maximum 52) plus the Numbers Score (maximum 11); the maximum overall education score was 115 and the minimum score was 0.

### Statistical analyses

Physical activity data were expressed as Accelerometer Units (AU)/min [18,19]. To address the primary hypothesis, ANOVA for physical activity/time was performed and post hoc 2-tailed, 2-sided paired t tests were used (JMP 9.0.3 statistical software, Cary, North Carolina).

Data were expressed as ± standard deviation, unless otherwise indicated, and statistical significance was defined as P < 0.05.

## Results

The demographics of the children are shown in Table 1. For the intervention classroom, 13 out of 17 (76%) children in the class agreed to enter the study. For the control classroom, 12 out of 16 (75%) children agreed to enter the study.

The two groups were well matched for age and race. All the children tolerated the study protocol without injury or complaint.

Body weight and height at baseline and after the eight-week intervention are shown in Table 2. As expected, weight and height increased significantly over the 8-week study for both the intervention and control groups (the children were growing). When the intervention and control groups were compared, between-group changes were not significantly different.

**Figure 1:**
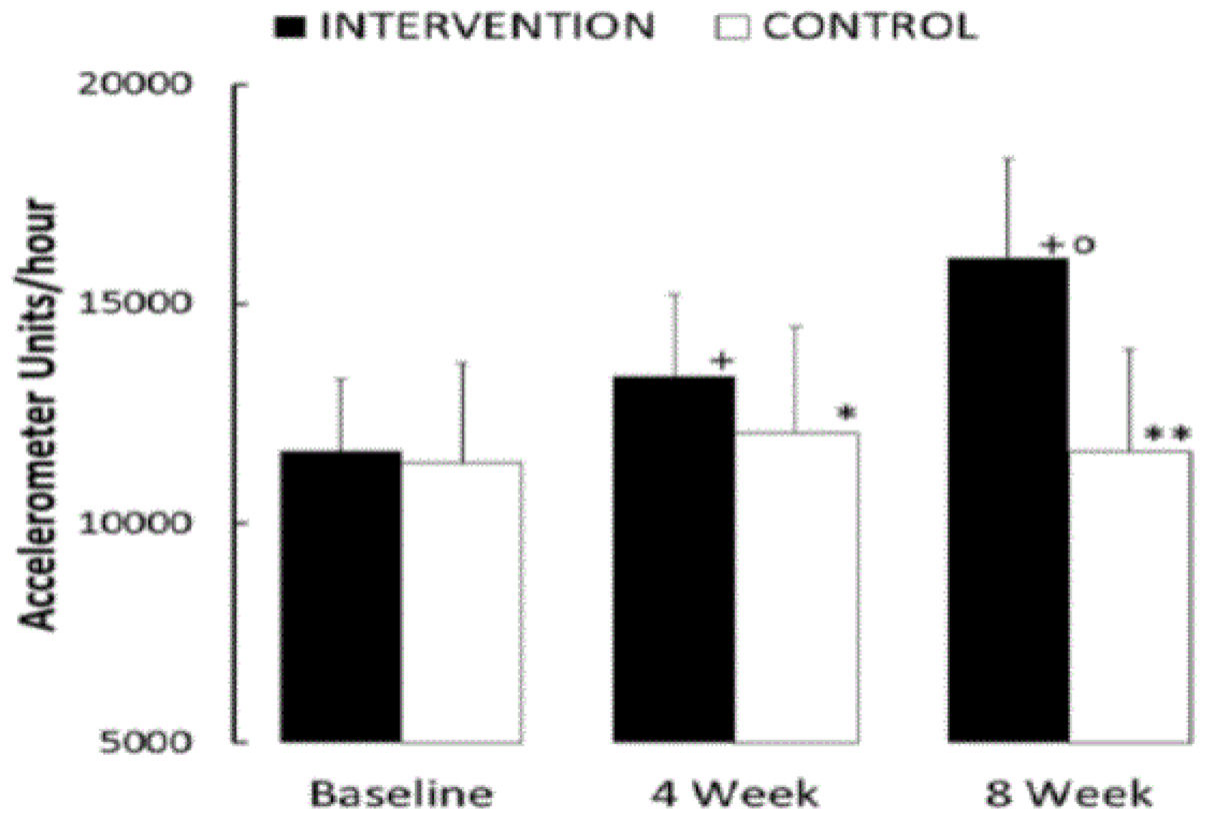
School Day Physical Activity for Intervention and Control Groups. School day physical activity data were compared with baseline for when preschool students received an Intervention of three 5-minute activity-promoting tasks/ day for 8 weeks. These data were compared to those of children who did not receive the activity-promoting intervention (Control). Data are shown as Accelerometer Units/hour + Standard Deviation. Comparison of the Intervention and Control groups ^*^ P = 0.02, ^**^ P < 0.001. Comparison with Baseline: + P < 0.001. Comparison Week 8 versus Week 4; ^O^ P < 0.001.

The hypothesis was that preschool children exposed to 3 five-minute activity breaks per day would increase their overall school time physical activity compared to a control group not receiving the intervention. The hypothesis was supported by the data (Figure 1) and the null hypothesis (of no change) was rejected.

After the 8-week intervention, children in the intervention group increased their school time activity from 11,641 ± (SD) 1,368 Acceleration Units (AU)/hour to 16,058 ± 2,253 AU/hour (P < 0.001).

The children in the control group did not increase their school time daily activity (11,379 ± 2,427 cf 11,624 ± 2,441; ns). The mean increase for the Intervention Group over the eight weeks was 4,417 ± 1,550 AU/hour, whereas for the Control Group 245 ± 1,091 AU/hour; this difference was statistically significant (P < 0.001).

We also examined mid-point accelerometer data (4 weeks of Intervention/Control) (Figure 1). After four weeks, there was a robust response to the intervention. Accelerometer data for the Intervention Group showed an increase of 1,702 ± 1,064 AU/hour versus the Control Group, 677 ± 821 AU/hour.

It should also be noted that the children in the control group were significantly more active in Week 4 than at baseline (P = 0.02), although this change was reversed by Week 8 (Figure 1).

The data suggest that in this study, children moved more when they were exposed to an intervention of three 5-minute activity breaks per school day compared to a control group that did not receive the intervention.

Was the increase in physical activity that we measured entirely associated with the three 5-minute activity breaks or did physical activity increase at other times as well? The data demonstrate the latter; namely that the intervention was associated with increased school time physical activity beyond the three 5-minute activity breaks.

Week 4 daily activity was 13,343 ± 2,113 AU/hour. At this point in time, the intervention was well established and was being applied in a constant fashion.

The fact that by Week 8, physical activity increased further to, 16,058 ± 2,253 AU/hour (P < 0.001) demonstrates that physical activity must have increased throughout the school day because the intervention did not change in the interim.

The secondary hypothesis was that children receiving the three 5-minute activity breaks per school day would show improved literacy and numeracy scores compared to the control group.

The data supported the secondary hypothesis, refuting the null hypothesis of no change.

Table 3 shows the education scores for the children in the Intervention and Control groups at Baseline and at Week 8.

In the Control Group, education scores improved overall which would be expected as the children were being educated. Students in the Intervention Group improved their education scores more than students in the Control Group (improvement of overall education score 18 ± 12 cf 8 ± 7 points, P = 0.01); Letter Recognition improved in particular (improvement of Letters Score 9 ± 6 cf 2 ± 4 points, P = 0.001). The children in the Intervention Group showed improved learning compared to the children in the Control Group.

The study supported both the primary and secondary hypotheses, that three 5-minute activity breaks were associated with a 40% increase in school time physical activity and improved learning in preschool children.

## Discussion

Only about one-half of preschool-age children meet physical activity guidelines [22] and leading authorities have suggested that practices be put in place to reverse this [22]. At the same time, funds are decreasingly available to help children be physically active both with respect to building activity-promoting spaces and supporting trained personnel to reverse this trend [23]. Here we tested an inexpensive approach to incorporating three 5-minute activity breaks into a preschool curriculum in a public rural school. The data demonstrate that this simple intervention was not only associated with substantial increases in school time physical activity but also, improved learning.

Many complex multifaceted interventions have been tried to improve the activity levels of young children [24]. However, many of these interventions require complex strategies and few are subject to stringent evaluation. The advantage of the three 5-minute break intervention we used is that it can be instantly implemented and costs <$1/child. What surprised us was the magnitude of the response in the children – a 40% increase in school-time physical activity at 8 weeks – far greater than accounted for by the bouts of activity alone. This is not the first study to examine the impact of short activity breaks in young children on learning. The consensus is that these types of breaks enhance learning [25] and we confirmed this in our study. Interestingly, a greater dose of activity would not be predicted to provide a greater improvement in learning [26].

School-based interventions in pre-school children are often poorly validated using untested techniques and uncontrolled study designs [24,26]. We applied a controlled design, robust technologies and established evaluation methodologies. This, in part, explains why the data so clearly demonstrate benefit for the intervention compared to the control group. If further studies replicate our findings and assign health benefit, this approach could be easily disseminated in many schools. The benefit of this approach is that special educators are not needed, the ‘equipment’ has almost no cost and time is not taken from education – in fact education improves.

We recognize that the study’s small size was a limitation, although the study was adequately powered to address our primary and secondary hypotheses. Nonetheless, it was too small to be broadly generalizable for several reasons. The school we studied was in a rural community and was able to provide a dedicated classroom for the intervention and control conditions; we cannot be sure that this intervention would be as successful in an urban environment. The ethnic composition of the children, although representative for the community we studied, may not be representative of other regions where ethnic composition may differ. An enthusiastic principal and willing teacher supported the intervention—in a school where this is not the case, we cannot be certain the intervention would have been as effective.

In conclusion, within the context of this experimental paradigm, it is feasible to deploy a low-cost, safe, and scalable approach to promote physical activity without specifically trained teachers or specialized equipment. Three 5-minute activity breaks in preschool children were associated with increased school time physical activity and improved learning.

## Figures and Tables

**Table 1:** Participant Demographics. Demographics of children enrolled in the low-cost and scalable classroom-based approach to promote physical activity in preschool children.

	Intervention Group	Control Group
n	13	12
Boys	9	5
Girls	4	7
3 year olds	2	1
4 year olds	9	8
5 year olds	2	3
White	11	11
Hispanic	2	0
African American	0	1

**Table 2:** Height and Weight of Intervention and Control Group at Baseline and after 8 Weeks. Height and weight of children enrolled in the low-cost and scalable classroom-based approach to promote physical activity in preschool children. Data are shown as mean ± SD for the students in the Intervention (n = 13) and the Control (n = 12) groups. Comparing Baseline versus Week 8, ^*^ P = 0.005, ^**^ P < 0.001). There were no statistically significant differences between the groups.

Baseline			Week 8		Change	
	Height(cm)	Weight (kg)	Height (cm)	Weight (kg)	Height (cm)	Weight (kg)
Intervention	112 ± 5.7	43.4 ± 6.8	114 ± 5.0	44.5 ± 6.2	1.62 ± 0.90 ^**^	1.08 ± 1.13 ^*^
Control	114 ± 4.6	46.9 ± 9.7	115 ± 4.5	48.5 ± 9.3	1.25 ± 0.62 ^**^	1.58 ± 0.79

**Table 3:** Education Scores of Intervention and Control Group at Baseline and after 8 Weeks. Education (Letters, Sounds and Numbers) Scores for children enrolled in the low-cost and scalable classroom-based approach to promote physical activity in preschool children. Data are shown as mean ± SD, for the students in the Intervention (n = 13) and the Control (n = 12) groups. Paired comparisons are shown for the Intervention and Control Groups comparing Week 8 with Baseline. The changes associated with Intervention and Control conditions were compared between the groups.

		Intervention	Baseline vs. Week 8	Control	Baseline vs. Week 8	Intervention vs. Control
Baseline	Letters	28 ± 13		36 ± 13		
	Sounds	26 ± 14		29 ± 11		
	Numbers	7.0 ± 3.7		7.9 ± 3.2		
	OVERALL	63 ± 32		72 ± 26		
Week 8	Letters	38 ± 10		37 ± 12		
	Sounds	32 ± 13		34 ± 12		
	Numbers	8.8 ± 2.5		8.8 ± 3.3		
	OVERALL	81 ± 25		80 ± 27		
Change	Letters	9.0 ± 5.6	P = 0.0002	1.5 ± 3.6		P = 0.001
	Sounds	6.8 ± 6.5	P = 0.003	5.3 ± 5.0	P = 0.004	
	Numbers	1.7 ± 2.0	P = 0.01	0.83 ± 0.84	P = 0.005	
	OVERALL	18 ± 12	P = 0.0002	8 ± 7	P = 0.003	P = 0.01
